# Where Is Pediatric Rheumatology Going, and Why a New Section of Frontiers in Pediatrics?

**DOI:** 10.3389/fped.2020.00306

**Published:** 2020-06-18

**Authors:** Rolando Cimaz

**Affiliations:** Department of Clinical Sciences and Community Health, University of Milan, Milan, Italy

**Keywords:** pediatric rheumatology, pediatric, rheumatology, arthritis, juvenile

Frontiers in Pediatrics has asked me to be Chief Editor for the new Pediatric Rheumatology section, and I enthusiastically accepted this new adventure. Pediatric rheumatology had long been a neglected specialty, but is now receiving worldwide attention. It has gathered the interest of hundreds of physicians and health professionals alike, who are involved in both the clinical part of the work and in all the other research and educational activities that are connected to the field.

This is why I chose to write this Grand Challenge on where the specialty came from and where we think it will go.

Why is this new subsection important for one, our European meetings were originally only being attended by a few dozen people, and now the Pediatric Rheumatology European Society (PReS) meetings attract a crowd of almost 1,500. Moreover, the specialty that was once limited to North America and some European nations has grown worldwide, with 91 countries included in the Pediatric Rheumatology International Trials Organization, and many activities from other organizations, such as the North American CARRA and PRSCG. The interest for clinicians and clinician-scientists alike is growing at a fast pace, and the American College of Rheumatology has set up programs for pediatric residents that are interested in our specialty and hopefully will pursue a career studying inflammatory diseases in children and adolescents.

Originally, acute rheumatic fever and juvenile rheumatoid/chronic arthritis were the main topics that junior doctors encountered and studied. However, all other chronic inflammatory disorders, such as all the juvenile forms of connective tissue disorders (lupus, systemic sclerosis, vasculitis, dermatomyositis, etc.) are now researched as part of the field as well. The field of interest has subsequently expanded exponentially, with many other disorders becoming of interest for pediatric rheumatologists, either from a clinical standpoint or for scientific study. Now the range of diseases in our specialty includes not only inflammatory disorders, but also non-inflammatory forms such as genetic and metabolic arthropathies, chronic pain syndromes, bone dysplasia, and so on. Imaging is another topic that deserves special attention due to its many new findings, with rheumatologists now needing to be knowledgeable about the diagnostic possibilities of old tools, e.g., ultrasound, and of new technical advancements such as the powerful MRI machines which have proven valuable for both clinical and research use. The list of novel actors in our specialty continues with the involvement of organ systems previously not considered a matter for rheumatologists. This includes uveitis, that nowadays is treated more by us than by ophthalmologists, who are not very familiar with the potent medications that have fortunately changed the outcome of our severely affected patients! I should also mention the recently discovered field of autoinflammatory disorders. These disorders are at the crossroads of immunology and rheumatology, and the collaboration between our two specialties is shedding new light on the obscure pathogenesis of these disorders, and helping in the identification of new treatment targets.

It is clear that things have changed a lot in the activities of our younger colleagues, who can start their career with a choice of different scientific paths. These will hopefully include residents and fellows alike, as well as PhD students. We now have excellent scientists who practice pediatric rheumatology as well, unlike in the past. Laboratory research has open questions for almost all of our diseases now, and this has helped not only to draw more fellows and residents to the field, but also to advance basic scientific knowledge and help find the cure for many severe disorders. These two aspects are now more and more intermingled, and in recent years we have more evidence of the reciprocal advantages of bench and bedside. For example, the classification of patients according to the response to treatment has brought advantages to both clinical outcomes and to knowledge of disease pathogenesis with regard to systemic onset JIA. Response to IL-1 inhibition seems to be a peculiar characteristic to both systemic JIA and autoinflammatory disorders, and this concept came well before the market authorization of anti-IL-1 drugs (1). The inclusion of systemic JIA in the spectrum of autoinflammatory diseases rather than in the traditional JIA umbrella term is therefore possible in future classification systems. New players in the field, such as type I interferon, are now being considered, and the so-called interferonopathies are now a well-known reality, about which we are still learning but that from obscure and unknown diseases have now in some cases become identifiable with genetic analyses (2). Again, research has helped to bring some hope to these unfortunate children, with the use of Jak-inhibitors. Finally, many of the biologicals used for rheumatoid arthritis have also been approved for JIA, and this has become standard treatment for refractory cases (3, 4). The off-label use of TNF inhibitors and other anti-cytokines is increasingly needed, especially for orphan diseases which are common in pediatric rheumatology clinics.

The specialty of pediatric rheumatology is thriving in science, as demonstrated by the rising number of grant applications, and by the excellent results obtained by winners of European grants such as Share, Pharmachild, or Diamonds, to mention a few. Transnational collaboration across countries and continents has achieved great results for clinicians and researchers, and all of this progress will ultimately benefit our patients.

Now many countries have their own national pediatric rheumatology society, both in Europe and across other continents including America, Asia, and, more recently, Africa. PReS has its own meeting, this year in its 26th edition, and the original Park City Meeting (subsequently moved to warmer areas!) is now also sponsored by the ACR. There are several high ranked general rheumatology journals that have specific sections for pediatric rheumatology, but only one other journal (Pediatric Rheumatology, formerly known as PROJ) is devoted specifically to our specialty.

We think that time to expand has come, and look forward to all proposals! See you soon with the first Golden Research Topic, which I hope you will enjoy.

## Author Contributions

The author confirms being the sole contributor of this work and has approved it for publication.

## Conflict of Interest

The author declares that the research was conducted in the absence of any commercial or financial relationships that could be construed as a potential conflict of interest.
